# Supplementary material for: Artificial intelligence-based classification of Spitz tumors
Source: J Pathol Inform. 2026 Feb 16;21:100650. doi: 10.1016/j.jpi.2026.100650 (PMC12989957; doi:10.1016/j.jpi.2026.100650)
Supplement: Supplementary material [file mmc1.pdf]

Supplementary Material

Supplementary Table 1: Results for Spitz genetic aberration prediction using the logistic regression model with clinical features only.

Metric	Classes	Performance
Accuracy (95% CI)	*ALK*, *ROS1*, *NTRK*, other	0.22 (0.14-0.30)
AUROC (95% CI)	*ALK* vs. rest	0.61 (0.44-0.77)
	ROS1 vs. rest	0.50 (0.38-0.62)
	NTRK vs. rest	0.39 (0.28-0.51)
	Other vs. rest	0.56 (0.41-0.70)

vs. = versus

Supplementary Table 2: Results for Spitz genetic aberration prediction using the image-only AI model based on features extracted with the first stage of HIPT.

Metric	Classes	Performance	
		Internal WSIs	Consultation WSIs
Accuracy (95% CI)	*ALK*, *ROS1*, *NTRK*, other	0.30 (0.22-0.38)	0.29 (0.23-0.35)
AUROC (95% CI)	*ALK* vs. rest	0.66 (0.50-0.81)	0.54 (0.38-0.68)
	ROS1 vs. rest	0.50 (0.37-0.62)	0.57 (0.44-0.69)
	NTRK vs. rest	0.64 (0.52-0.76)	0.62 (0.51-0.73)
	Other vs. rest	0.60 (0.46-0.72)	0.57 (0.44-0.70)

vs. = versus

Supplementary Table 3: Results for Spitz genetic aberration prediction using the image-only AI model based on features extracted with the second stage of HIPT.

Metric	Classes	Performance	
		Internal WSIs	Consultation WSIs
Accuracy (95% CI)	*ALK*, *ROS1*, *NTRK*, other	0.39 (0.30-0.49)	0.36 (0.28-0.46)
AUROC (95% CI)	*ALK* vs. rest	0.67 (0.53-0.80)	0.56 (0.40-0.72)
	ROS1 vs. rest	0.63 (0.51-0.74)	0.67 (0.54-0.79)
	NTRK vs. rest	0.68 (0.56-0.79)	0.67 (0.55-0.78)
	Other vs. rest	0.70 (0.58-0.82)	0.66 (0.52-0.78)

vs. = versus

Supplementary Table 4: Results for Spitz diagnostic classification prediction using the logistic regression model with clinical features only.

Metric	Classes	Performance
Accuracy (95% CI)	Benign, Intermediate, Malignant	0.35 (0.26-0.45)
AUROC (95% CI)	Benign vs. rest	0.44 (0.32-0.56)
	Intermediate vs. rest	0.63 (0.52-0.73)
	Malignant vs. rest	0.52 (0.35-0.68)

vs. = versus

Supplementary Table 5: Results for Spitz diagnostic classification prediction using the image-only AI model based on features extracted with the first stage of HIPT.

Metric	Classes	Performance	
		Internal WSIs	Consultation WSIs
Accuracy (95% CI)	Benign, Intermediate, Malignant	0.48 (0.37-0.58)	0.41 (0.32-0.52)
AUROC (95% CI)	Benign vs. rest	0.63 (0.52-0.74)	0.64 (0.52-0.74)
	Intermediate vs. rest	0.52 (0.40-0.63)	0.53 (0.41-0.65)
	Malignant vs. rest	0.72 (0.59-0.83)	0.73 (0.59-0.84)

vs. = versus

Supplementary Table 6: Results for Spitz diagnostic classification prediction using the image-only AI model based on features extracted with the second stage of HIPT.

Metric	Classes	Performance	
		Internal WSIs	Consultation WSIs
Accuracy (95% CI)	Benign, Intermediate, Malignant	0.50 (0.39-0.60)	0.46 (0.36-0.55)
AUROC (95% CI)	Benign vs. rest	0.66 (0.55-0.76)	0.68 (0.57-0.78)
	Intermediate vs. rest	0.56 (0.44-0.67)	0.62 (0.51-0.73)
	Malignant vs. rest	0.71 (0.55-0.85)	0.79 (0.68-0.89)

vs. = versus

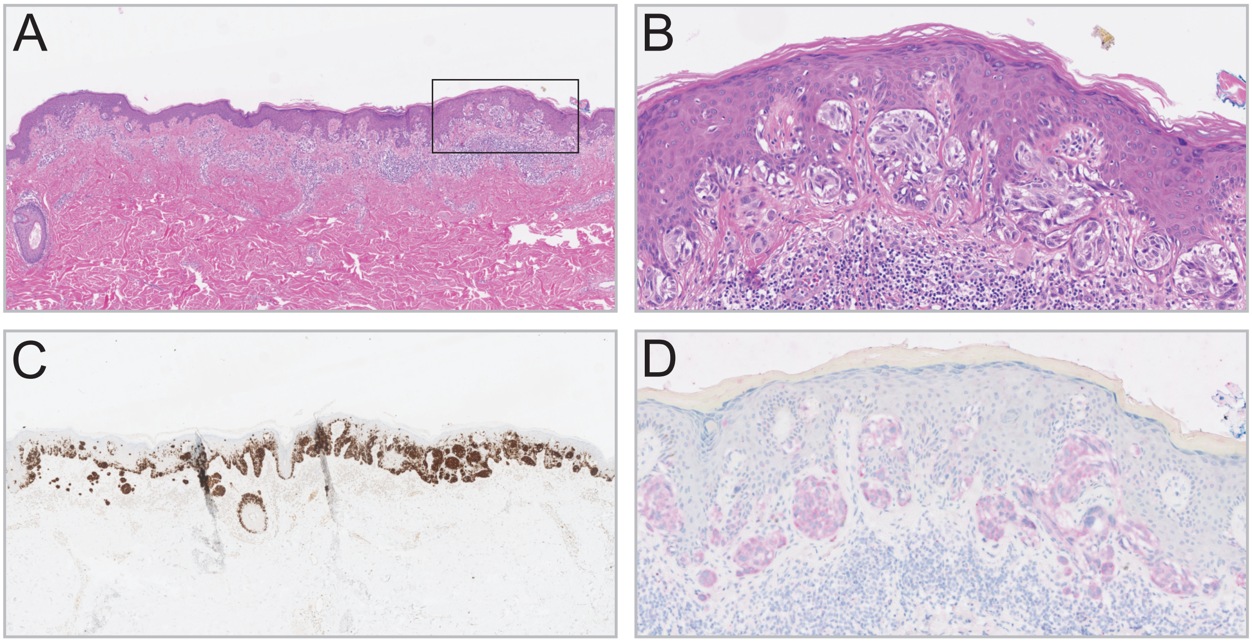

Supplementary Figure 1: Spitz nevus with a *ROS1* fusion from the back of a 59-year-old man. The lesion was incorrectly classified as a conventional melanoma by 3 out of 4 pathologists in the reader study based on the histology on H&E and clinical information. The AI model also incorrectly classified the lesion as a conventional melanoma based only on the H&E-stained histology. The lesion showed co-expression of p21 and p16 and weak to no expression of PRAME. No *TERT* promoter mutation was found using NGS. SNP array analysis was not performed because the tumor cell percentage was estimated to be too low for reliable results. (A) Overview of the Spitz tumor. (B) Part of the lesion at a higher magnification. (C) Melan-A highlights the architecture of the lesion. (D) Positive staining for ROS1.

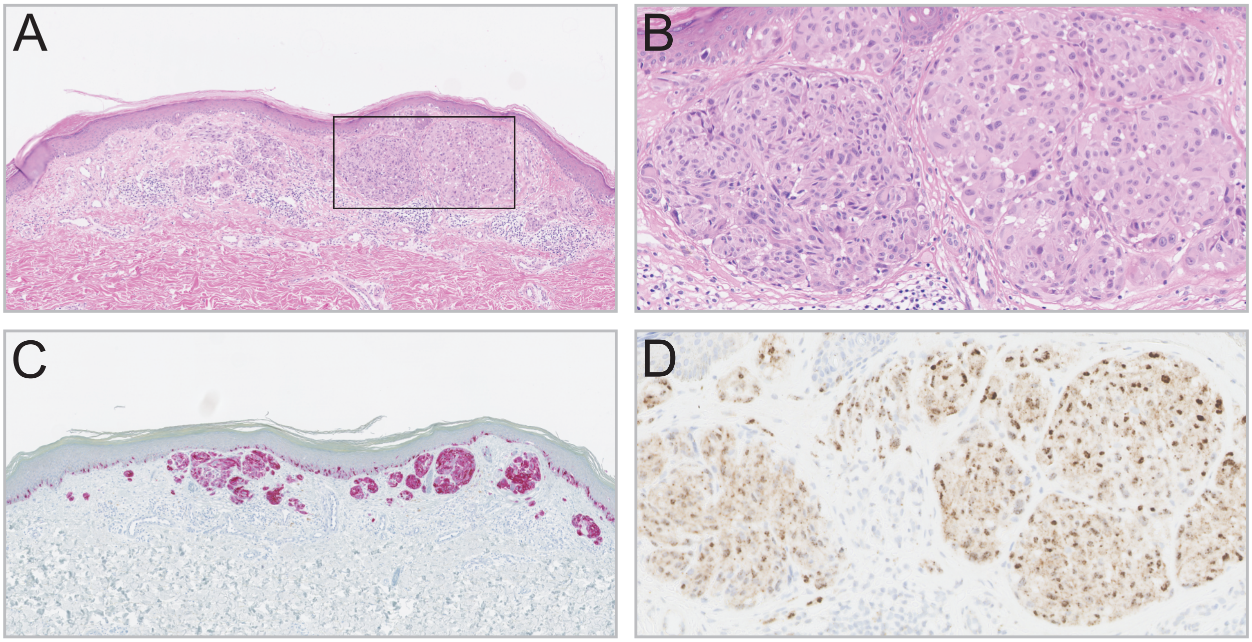

Supplementary Figure 2: High-grade Spitz melanocytoma with an *NTRK3* fusion from the lower leg of a 25-year-old woman. The lesion was incorrectly classified as a conventional melanoma by 3 out of 4 pathologists in the reader study based on the histology on H&E and clinical information. The AI model correctly classified the lesion as a Spitz tumor based only on the H&E-stained histology. The lesion showed slight loss of p16 expression and expression of PRAME in approximately 10% of the lesion cells. No *TERT* promoter mutation was found using NGS. A trisomy of chromosome 15 was found using SNP array analysis, but further assessment of copy number variations was not possible due to the low tumor cell percentage. An *NTRK3* fusion was found using Archer. (A) Overview of the Spitz tumor. (B) Parts of the lesion at a higher magnification. (C) Melan-A highlights the architecture of the lesion. (D) Positive staining for NTRK.

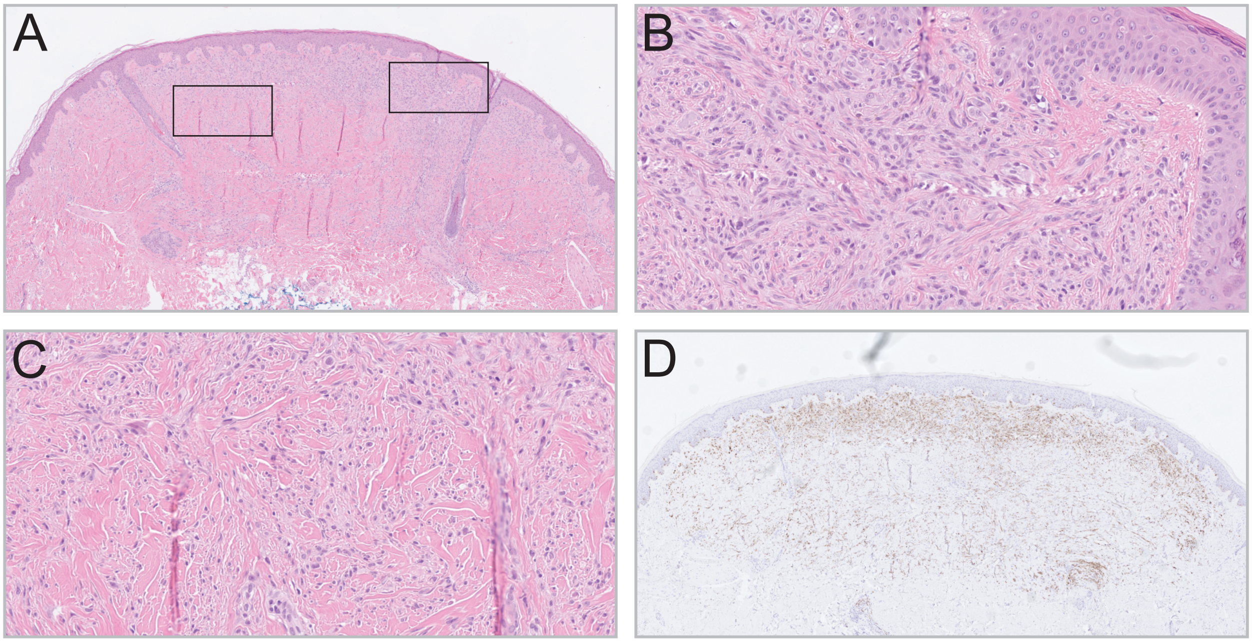

Supplementary Figure 3: High-grade Spitz melanocytoma with an *HRAS* mutation from the upper arm of a 50-year-old woman. The lesion was correctly classified as a Spitz tumor by 4 out of 4 pathologists in the reader study based on the histology on H&E and clinical information. The AI model incorrectly classified the lesion as a conventional melanoma based only on the H&E-stained histology. The lesion showed normal p16 expression and no expression of PRAME. An *HRAS* mutation and no *TERT* promoter mutation were found using NGS. A total of 6 copy number variations (partial loss of chromosome 1p, partial loss (2×) and partial gain of chromosome 7q, loss of chromosome 8, and partial gain of chromosome 11p) were found using SNP array analysis. (A) Overview of the Spitz tumor. (B, C) Parts of the lesion at a higher magnification. (D) Melan-A highlights the architecture of the lesion.

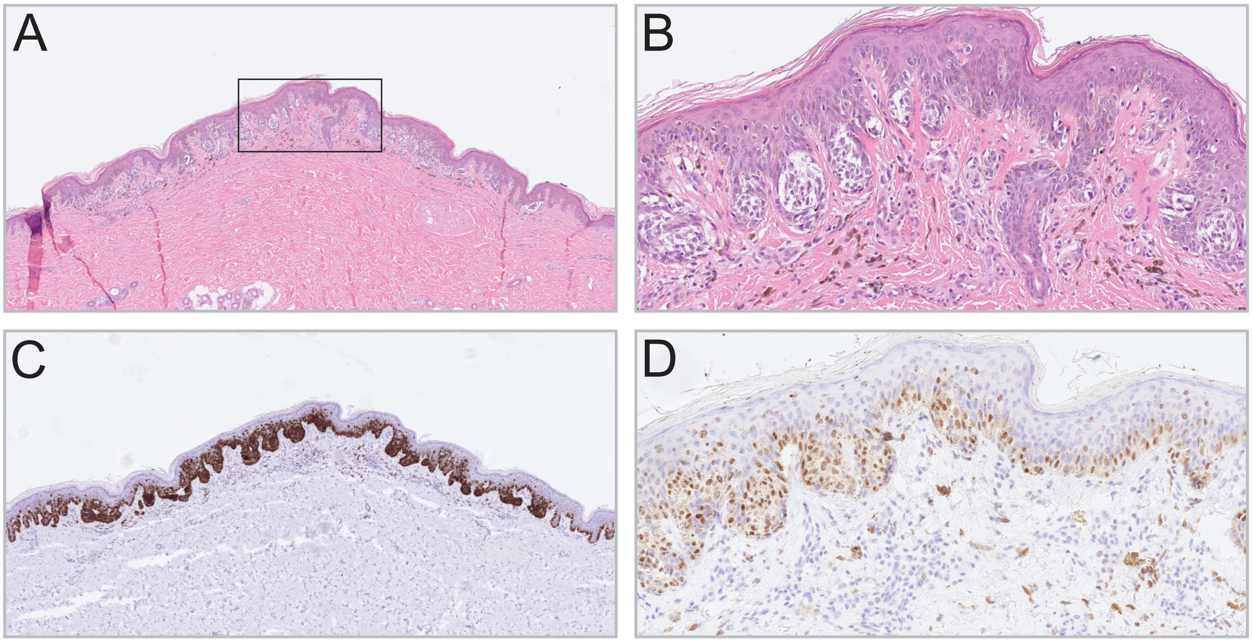

Supplementary Figure 4: Conventional melanoma with an uncommon combination of a *BRAF* and *NRAS* mutation from the upper leg of a 38-year-old woman. The lesion was incorrectly classified as a Spitz tumor by 3 out of 4 pathologists in the reader study based on the histology on H&E and clinical information. The AI model also incorrectly classified the lesion as a Spitz tumor based only on the H&E-stained histology. The lesion showed slight expression of p16 and expression of PRAME in part of the lesion. An activating *NRAS* mutation, a low-frequency inactivating *BRAF* mutation, and no *TERT* promoter mutation were found using NGS. A total of 6 copy number variations (gain of chromosome 1p, partial gain of chromosome 6p, partial loss of chromosome 6q, partial loss of chromosome 8p, gain of chromosome 8q, and a trisomy of chromosome 20) were found using SNP array analysis. (A) Overview of the conventional melanoma. (B) Part of the lesion at a higher magnification. (C) Melan-A highlights the architecture of the lesion. (D) Positive straining for PRAME.

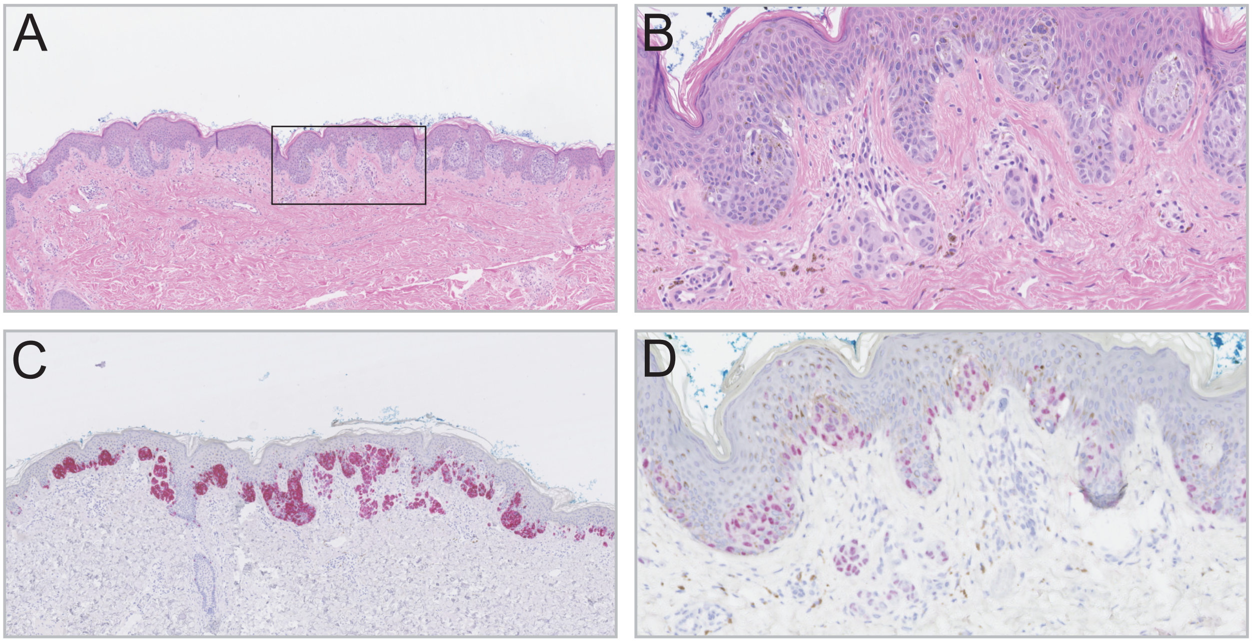

Supplementary Figure 5: Conventional melanoma with a *BRAF* mutation from the upper arm of a 42-year-old woman. The lesion was incorrectly classified as a Spitz tumor by 4 out of 4 pathologists in the reader study based on the histology on H&E and clinical information. The AI model correctly classified the lesion as a conventional melanoma based only on the H&E-stained histology. The lesion showed normal expression of p16 and strong expression of PRAME. A low-frequency *BRAF* (non-V600E) mutation and a *TERT* promoter mutation were found using NGS. No copy number variations were found using SNP array analysis, although this could be due to the low tumor cell percentage, which was estimated at less than 10%. (A) Overview of the conventional melanoma. (B) Part of the lesion at a higher magnification. (C) Melan-A highlights the architecture of the lesion. (D) Positive straining for PRAME.

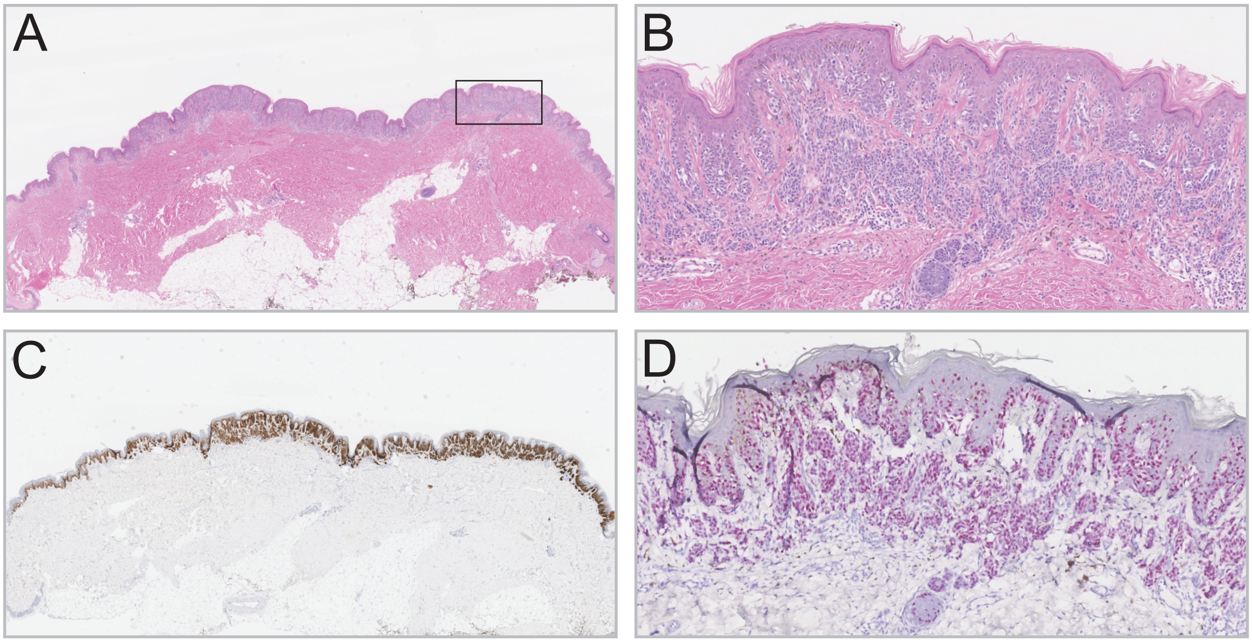

Supplementary Figure 6: Conventional melanoma with a *BRAF* mutation from the upper leg of a 49-year-old woman. The lesion was correctly classified as a conventional melanoma by 4 out of 4 pathologists in the reader study based on the histology on H&E and clinical information. The AI model incorrectly classified the lesion as a Spitz tumor based only on the H&E-stained histology. The lesion showed loss of p16 expression and expression of PRAME in more than 75% of the lesion cells. A *BRAF* mutation and no *TERT* promoter mutation were found using NGS. A total of 6 copy number variations (gain of chromosome 6p, gain of chromosome 8q, loss of chromosome 8p, loss of chromosome 9p (including heterozygous loss of *CDKN2A*), loss of chromosome 16q, and partial loss of chromosome 12q) were found using SNP array analysis. (A) Overview of the conventional melanoma. (B) Part of the lesion at a higher magnification. (C) Melan-A highlights the architecture of the lesion. (D) Positive straining for PRAME.
